# Parents of early‐maturing girls die younger

**DOI:** 10.1111/eva.12780

**Published:** 2019-03-23

**Authors:** Peeter Hõrak, Markus Valge, Krista Fischer, Reedik Mägi, Tanel Kaart

**Affiliations:** ^1^ Department of Zoology University of Tartu Tartu Estonia; ^2^ Estonian Genome Center University of Tartu Tartu Estonia; ^3^ Institute of Veterinary Medicine and Animal Sciences Estonian University of Life Sciences Tartu Estonia

**Keywords:** age at death, age at menarche, breast development, heritability, human life‐history evolution, lifespan, pace‐of‐life syndrome, sexual maturation

## Abstract

According to the life‐history theory, rates of sexual maturation have coevolved with mortality rates so that individuals who mature faster tend to die younger. We used two data sets, providing different markers for the speed of pubertal development to test whether rates of sexual maturation of women predict the age at death of their parents. In the data set of Estonian schoolgirls born between 1936 and 1961, the rate of breast development predicted lifespan of both mothers and fathers (irrespectively of their socio‐economic position), so that parents of rapidly maturing girls died at younger age. This finding supports the view that fast maturation rates in humans have coevolved with short lifespans and that such trade‐offs can be detected as intergenerational phenotypic correlations in modern populations. Menarcheal age of participants of Estonian Biobank (born between 1925 and 1996) did not predict the age of death of their mothers; however, it did predict survival of their fathers, but only in environment where the genetic variation is exposed (families where at least one parent had tertiary education). In such families (where girls also matured 0.2–0.4 years earlier than in poorly educated families), 1‐year delay in daughter's menarche corresponded to 9% lower hazard of father's death. Heritability of menarcheal age was also highest in well‐educated families. The latter findings are consistent with the idea that genetic differences in the rate of pubertal maturation may be expressed most clearly in well‐off families because in such families, the contribution of environmental variance to total phenotypic variance in menarcheal age is smallest. Our findings suggest that with global improvement and equalization of growth conditions, reductions of environmental variation in the rate of maturation increasingly expose the genetic differences in menarcheal age to selection. Under such conditions, selection on menarcheal age has a potential to affect the evolution of lifespan.

## 1 INTRODUCTION

Rate of sexual maturation in humans has remarkable genetic component with heritability estimates for different measures ranging from 0.44 to 0.95 (Ge, Natsuaki, Neiderhiser, & Reiss, 2007; Hodges & Palmert, 2007; Towne et al., 2005). A common evolutionary explanation to preservation of heritable variation in such life‐history traits is pleiotropic linkage with other components of fitness (Stearns, 1989). For instance, early menarche has both evolutionary benefits (enabling to start reproduction earlier) and costs, such as those imposed by trade‐offs between rates of pubertal maturation versus somatic growth and cognitive/psychosocial development (Mendle, Turkheimer, & Emery, 2007). According to the life‐history theory, rates of maturation and reproduction should evolve to track the variation in mortality rates. Children inheriting from their parents a propensity to die early are thus supposed to have evolved a predisposition for faster sexual maturation. Therefore, measures of pubertal development appear potentially informative markers of life‐history speed that can be used for quantifying trade‐offs between maturation and longevity, particularly so in societies where age of first reproduction is decoupled from the age of obtaining sexual maturity (Gagnon, 2015).

Consistent with this life‐history trade‐off are the findings that at phenotypic level, early menarche associates with increased risk of all‐cause mortality, ischaemic heart disease and breast cancer (Charalampopoulos, McLoughlin, Elks, & Ong, 2014). Recent genome‐wide association studies (GWAS) have identified that genomic loci associated with menarcheal age substantially overlap with genes implicated in various (particularly metabolic) diseases (Perry et al., 2014) and age of first intercourse (Day et al., 2016). Further, genetic variants that delay menarche were found to associate with longer lifespan of both mothers and fathers of participants of the UK and US biobanks (Mostafavi et al., 2017).

Although consistent with the life‐history theory, these findings leave open the question of whether and under what conditions the observed genetic correlations between rates of maturation and lifespan are currently exposed to selection in the phenotype. If a negative genetic correlation between two traits exists but the population is experiencing environmental conditions in which most of the variation within and between the two traits is environmentally induced, the genetic differences are not expressed in the phenotypes and, thus, remain inaccessible for the natural selection (Stearns, 1989). One possibility to test whether the particular combinations of maturation rates and lifespans that are characteristic to fast versus slow life‐histories are expressed in the phenotypes would be demonstration of intergenerational associations between longevity and rate of sexual maturation (Hõrak, 2017).

Here, we use two independent data sets, providing different markers for the speed of pubertal development to test whether rates of sexual maturation of women predict the age at death of their parents. The first data set relies on menarcheal age of participants of Estonian Biobank (*n* = 17,280, born between 1925 and 1996, average = 1,970.5 ± 13.9 [*SD*]). This data set had number of sib and mother–sister pairs, thus permitting assessment of heritabilities and genetic and environmental variances in menarcheal age. The second data set relies on the rate of breast development (see Marshall & Tanner, 1969), recorded in an extensive study of Estonian schoolchildren (*n* = 9,331, born between 1936 and 1961, average = 1,948.0 ± 4.6), performed by Prof. Juhan Aul between 1956 and 1969 (see Hõrak & Valge, ). This data set does not enable genetic analyses; however, its advantage is that 97% of parents of participants are dead, yielding extremely precise estimates for parental lifespan.

To account for environmentally induced variation in pubertal development, we stratified our samples according to the measures of the quality of home environment. In the case of Estonian Biobank, we used highest level of education obtained by either mother or father as a proxy of resource availability for participants during growth (see Mendle, Moore, Briley, & Harden, 2016). As inadequate nutrition and health care and imposed physical workload delay menarche (Sohn, 2017), we expected that such environmental constraints will be ameliorated among women growing up in most highly educated families. The rationale behind this reasoning is that genetic between‐individual differences in quantitative trait expression become proportionally larger when environmental effects on trait expression decrease (Bolund, Hayward, Pettay, & Lummaa, 2015; Hoffmann & Merilä, 1999). In case of women growing up in well‐educated families, we expect that the contribution of environmental variance to total phenotypic variance in menarcheal age is smaller (due to higher proportion of individuals receiving proper nutrition and health care and less workload) than among the women growing up in less well‐educated families that are more likely to experience socio‐economic deprivation. For instance, a woman with a genetic predisposition for early menarche will not mature early under conditions of resource limitation but would do so (i.e., realize her genetic potential for early maturation) when growing up under conditions where access to resources is not limited. In the data set of schoolgirls, we relied on the similar reasoning; however, in this case we used measures of parental socio‐economic position (SEP) to assess the quality of home environment due to incompleteness of record on parental educational attainment.

We will test whether our two markers of the rate of sexual maturation of women, measured under different settings, predict parental lifespan and whether this relationship depended on the quality of home environment, assessed on the basis of parental education or SEP. We predict that the association between daughters’ rate of sexual development and parental lifespan will be manifested contingent upon interaction with the quality of home environment, so that the measures of puberty predict parental lifespan most strongly in well‐off families. This prediction is based on the assumption that among the women growing up in families of well‐educated parents or those in nonmanual professions, variation in the rate of sexual development is more likely to reflect genetic differences between individuals than among the women growing up in less well‐off families (Bolund et al., 2015; Hoffmann & Merilä, 1999; Ong, Ahmed, & Dunger, 2006). The data set of Estonian Biobank allows us to test this using an animal model of menarcheal age based on the pedigree to estimate the contribution of additive genetic and environmental variances to total phenotypic variance of the trait.

## 2 METHODS

### 2.1 Data sets: Estonian biobank and Aul's database

Data on menarcheal age (see Tables 1 and 2 and Supporting Information Table S1, ESM) were obtained from the Estonian Genome Center. Estonian Genome Center at the University of Tartu is a population‐based biobank that recruited a cohort of 51,756 participants, including adults from all counties in Estonia, accounting for approximately 5% of the Estonian adult population during the recruitment period. Recruitment was performed during the period from 2002 to 2012 (Leitsalu et al., 2014). At baseline, an extensive phenotype questionnaire (including questions about parental age at death and participant's age at menarche, with a precision of 1 year) was conducted together with a measurement panel. Data on highest parental education of participants were obtained from Estonian Population Registry (https://e-estonia.com/solutions/interoperability-services/population-registry/), based on self‐reported data from Estonian population Census in 2011 (https://www.stat.ee/phc2011).

**Table 1:** Effect of highest parental education on menarcheal age of their daughters, adjusted for covariates (data of Estonian Biobank)

Effect	*df*	*F*	*p*
Highest parental education	2, 17,274	13.2	<0.0001
Birth year	1, 17,274	81.4	<0.0001
Highest parental education x birth year	2, 17,274	12.9	<0.0001

**Table 2:** Descriptive statistics for menarcheal age (means and least square [LS] means, adjusted for covariates in Table 1) and birth years of participants according to the highest education level obtained by their parents (data of Estonian Biobank)

Highest parental education	*N*	Menarcheal age (years)			Birth year	
		Mean (*SE*)	Range	LS mean (*SE*)	Mean (*SE*)	Range
Primary	5,628	13.60 (0.02)	8−19	13.42 (0.03)	1959.8 (0.16)	1925−1993
Secondary	8,651	13.28 (0.02)	9−19	13.32 (0.02)	1974.9 (0.13)	1931−1995
Tertiary	3,001	13.15 (0.03)	9−19	13.17 (0.03)	1977.8 (0.19)	1929−1996

Data on the rate of breast development were obtained from the anthropometric study performed by Juhan Aul between 1956 and 1969 (henceforth, Aul's database). The historical background of this sample is described by Hõrak and Valge (2015a). The data set involves 9,331 7‐year‐old to 24‐year‐old (mean = 14. 5, *SD *= 2.2) schoolgirls born between 1936 and 1961 (see Supporting Information Table S2 in ESM for the characteristics of their parents). Development stage of breasts was assessed on the basis of six‐point scale (0–5) by a single person; in the analyses, residuals from linear regression of breast development score on measurement age were used (the correlation between the breast development score and age at measurement was 0.74, *n* = 9,331, *p* < 0.0001). On the basis of parental professions recorded during data collection, parents of participants were assigned SEP values as unskilled manual workers, skilled manual workers and nonmanual workers. Data on parental lifespans were obtained from the Estonian Population Registry. Data processing was carried on anonymously under the licence of the Research Ethics Committee of the University of Tartu (protocol no. 275/T‐1, issued on 20 November 2017).

### 2.2 Data analyses

To verify that parental education is a suitable proxy for growth conditions in the Estonian Biobank data, we tested the effect of highest level of education obtained by either mother or father (factor with three levels corresponding to primary, secondary and tertiary education) in an ANCOVA model adjusting for the birth year of participants (to account for secular trend in menarcheal age) and its interactions with parental education in order to test whether the secular trend in menarcheal age differed between women originating from poorly versus highly educated families (Table 1). Additionally, we run the same model adjusting also for the height and body mass of participants (Supporting Information Tables S3 and S4 in ESM) as heavier and shorter women reach menarcheal age faster (Perry et al., 2014). The rationale of using highest parental education instead of mother's and father's education separately was increasing test power: for the subset of data that was available for analyses presented in Table 5, we had 17,271 observations for the highest parental education and only 9,362 observations for families for which the educational level of both parents was known. Survival analyses involving mothers’ and fathers’ education simultaneously are presented in Supporting Information Table S6 in ESM. This analysis involved only 1,011 dead mothers and 2,167 dead fathers, indicating that test power was substantially lower than in the analyses presented in Table 4. Effect of highest parental SEP on the rate of breast development in the Aul's data set was tested in the ANCOVA model adjusting for the age of measurement (Table 3).

**Table 3:** Test for the effects of parental SEP and birth date on daughters’ rate of sexual maturation (breast development in 6‐point scale; Aul's database)

Effect	*df*	*F*	*p*
Highest parental SEP	2, 9,326	16.4	<0.0001
Age at measurement	1, 9,326	8,450.4	<0.0001
Birth date	1, 9,326	1.2	0.270

Associations between daughters’ age at menarche, rate of breast development and survival of their parents were analysed in Cox proportional hazard models, using mother's or father's birth year as a covariate in order to adjust for secular trends in lifespan. Interaction between daughters’ rate of pubertal maturation and highest parental education level or SEP was also tested in the same models (Tables 5 and 6) because we assumed that associations between daughters’ rate of sexual maturation and parental lifespan would be the strongest among girls growing up in favourable conditions, that is, in families where at least one parent had tertiary education or was nonmanual worker. In the data set of Estonian Biobank, we limited the sample of fathers to those younger than 91 years because the data subset of women from highly educated families and late menarche (age = 15, fourth quantile) contained only one observation of a father surviving above 91 years. Descriptive statistics for the data used in the survival analyses in Tables 4, 5, 6 are presented in the Supporting Information Tables S1 and S2 in the ESM. 5,628 women with known menarcheal age had relatives with 50% or higher proportion of genome shared (IBD). To test whether this nonindependence of observations would affect our results, we reran the survival analyses retaining only one randomly selected sister per sibship. The results were identical to these obtained in the full sample in terms of significant and nonsignificant effects (Supporting Information Table S7 in ESM).

**Table 4:** Estimates of the heritability and variance structure of menarcheal age (residuals from the regression to the year of birth, obtained from the whole data set) in relation to the highest parental education level in the family, calculated from the animal model, using package VCE 6.0.2. *V*_A_, additive genetic variance; *V*_R_, residual variance, which includes environmental effects, nonadditive genetic variance (dominance and epistatic) and error variance; *V*_P_, phenotypic variance (data of Estonian Biobank). Estimates calculated from the model that accounts for the fixed effect of birth year (instead of using residuals) are presented in ESM (Table S5)

Highest parental education	*N*	Mean (*SD*)	*h* ^2^ (*SE*)	*V*_A_ (*SE*)	*V*_R_ (*SE*)	*V*_P_
Primary	5,628	0.036 (1.510)	0.559 (0.076)	1.273 (0.178)	1.005 (0.171)	2.278
Secondary	8,651	−0.003 (1.409)	0.550 (0.058)	1.093 (0.119)	0.892 (0.114)	1.985
Tertiary	3,001	−0.083 (1.345)	0.607 (0.133)	1.103 (0.247)	0.712 (0.238)	1.814

**Table 5:** Cox proportional hazard models for survival of mothers and fathers in relation to menarcheal age of their daughters and its interaction with highest parental education level in the family (data of Estonian Biobank)

Predictor	Hazard ratio (95% CI)	*p*
A. Mothers’ survival, *n* = 15,381, number of deaths = 3,264		
Mothers’ year of birth	1.028 (1.024−1.032)	<0.0001
Daughters’ menarcheal age (A)	1.005 (0.977−1.033)	0.741
Highest parental education is secondary (B)*	0.705 (0.350−1.423)	0.330
Highest parental education is tertiary (C)*	1.074 (0.287−4.017)	0.916
A x B	1.002 (0.952−1.055)	0.937
A x C	0.953 (0.862−1.054)	0.348
B. Fathers’ survival, *n* = 15,210, number of deaths = 6,316		
Fathers’ year of birth	1.009 (1.007−1.011)	<0.0001
Daughters’ menarcheal age (A)	0.993 (0.971−1.015)	0.538
Highest parental education is secondary (B)*	0.531 (0.328−0.861)	0.010
Highest parental education is tertiary (C)*	1.407 (0.585−3.385)	0.446
A x B	1.030 (0.994−1.068)	0.100
A x C	0.923 (0.864−0.987)	0.019

Notes

Effect of daughter's menarcheal age on survival of their mothers remained nonsignificant (HR = 1.003, 95% CI = 0.980‐1.026, *z* = 0.216, *p* = 0.829) after dropping the interaction between “highest parental education” and daughters’ menarcheal age from the model.

* Compared with parents from families where both parents had primary education.

**Table 6:** Cox proportional hazard models for survival of mothers and fathers in relation to rate of sexual maturation (age‐standardized breast development score) of their daughters and highest parental SEP in the family (Aul's database)

Predictor	Hazard ratio (95% CI)	*p*
A. Mothers’ survival, *n* = 5,747, number of deaths = 5,503		
Mothers’ year of birth	1.005 (1.002−1.009)	0.005
Daughters’ breast development score	1.055 (1.015−1.096)	0.007
Highest SEP skilled manual*	0.935 (0.872−1.002)	0.058
Highest SEP nonmanual*	0.832 (0.779−0.888)	<0.0001
B. Fathers’ survival, *n* = 5,070, number of deaths = 5,030		
Fathers’ year of birth	1.009 (1.006−1.013)	<0.0001
Daughters’ breast development score	1.082 (1.040−1.129)	0.0001
Highest SEP skilled manual*	0.872 (0.811−0.938)	<0.0001
Highest SEP nonmanual*	0.731 (0.682−0.785)	<0.0001

Notes

Interaction terms between breast development score and parental SEP were nonsignificant both in the case of mothers (HR = 0.949 [0.864‐1.047] and 0.996 [0.906‐1.095]) and fathers (HR = 1.029 [0.932‐1.137] and 0.977 [0.888‐1.075]).

* Compared with parents on nonskilled manual professions.

### 2.3 Pedigree analysis

In order to identify relatives, Biobank participants have been analysed using Illumina genotyping arrays: (a) Global Screening Array (GSA, *N* = 33,277), (b) HumanCoreExome (CE, *N* = 7,832), (c) HumanOmniExpress (OMNI, *N* = 8,137) and (d) 370K (*N* = 2,640). If samples were genotyped with different arrays, the following order of beadchips was preferred: GSA, OMNI, 370K, CE. The final set of genotyped data contained 48,163 unique individuals. The genotype calling for the microarrays was performed using Illumina's GenomeStudio v2010.3 software. The genotype calls for rare variants on the GSA and CE arrays were corrected using the zCall software (version May 8th, 2012). After variant calling, the data were filtered using PLINK v.1.90 by sample (call rate >95%, no sex mismatches between phenotype and genotype data, heterozygosity < mean ± 3 *SE*) and by marker (HWE *p* > 1 × 10^−6^, call rate >95%, and for the GSA array additionally by Illumina GenomeStudio GenTrain score >0.6, Cluster Separation Score >0.4). For the IBD calculation, we used PLINK v1.90 software and for that data from different arrays were merged. Variants not present in all arrays and variants with minor allele frequency <1% were removed from the analysis. Secondly, LD pruning was performed using PLINK command line option “‐‐indep‐pairwise 100 10 0.1” to remove variants from pairs, which had pairwise *r* ^2^ > 0.1. Lastly, IBD was calculated using 61,044 noncorrelated autosomal variants in PLINK with “—genome” command line option.

The pedigree was constructed based on information about sisters and mother–daughter pairs with known menarcheal age. There were in total 2,523 mother–daughter pairs, 638 pairs of sisters (including dizygotic twins), 32 triplets and one quartet of sisters, and seven pairs of monozygotic twins and one monozygotic triplet. To consider these genetic relationships in form of pedigree data, for each sisters’ group unique mother's and father's identification codes were generated (Supporting Information Figure S1A in ESM), in case of mother–daughter pairs the mother's unique identification code present in menarcheal age database was also used in pedigree file (Supporting Information Figure S1B), and in case of monozygotic twins and triplets, the same unique identification code was used for both twins and triplets (the menarcheal ages of sisters were considered as repeated measures of the same individual). As for some families menarcheal ages of three generations of women were known and the sisters were present also in different generations (Supporting Information Figure S1C), the maximum depth of pedigree was four generations (Supporting Information Figure S1D).

To assess the heritability of menarcheal age, we calculated its variance components from the pedigree data using the VCE 6.0.2 software package, which provides estimates of heritability (*h* ^2^), additive genetic, phenotypic and residual variance (Groeneveld, Kovac, & Mielenz, 2010). The software uses a restricted maximum‐likelihood (REML) approach, based on the animal model, in which individual's phenotype is broken down into its components of additive genetic value and other random and fixed effects. The pedigree was constructed based on information about mother–daughter pairs and sisters with known menarcheal age, and consists 2,523 mother–daughter pairs, 638 pairs of sisters (including dizygotic twins), 32 triplets and one quartet of sisters, and seven pairs of monozygotic twins and one monozygotic triplet. For each parental educational level, separate models were fitted with common pedigree data. To account for secular trend in menarcheal age, residuals from linear regression of menarcheal age on date of birth were used (separately for each parental educational level) in the animal model. Additionally, we run the model with actual menarcheal age considering birth year as a fixed effect (Supporting Information Table S5 in ESM). The estimates of heritability and variance components differed minimally (Table 4 vs. Supporting Information Table S5). However, in case of more complicated multivariate models not used in present study, the results from models with pre‐adjusted variables (residuals from linear regression) were more stable compared with the results of models applied on actual values and considering birth year as a fixed effect (the estimation process converged more quickly, and the parameters’ estimates from models considering different combinations of dependent variables were more similar). Results from the model with pre‐adjusted menarcheal age (Table 4) are presented in the main text order to facilitate comparison of *h* ^2^ values with previous studies which have used the same approach (Towne et al., 2005).

## 3 RESULTS

We first verified that our measures of the rate of pubertal development were sensitive to growth conditions in our study population. Menarche was latest (13.4 ± 0.03 years, *n* = 5,628) among the women growing up in families where both parents had primary education, preceded by the women from families where at least one parent had secondary (13.3 ± 0.02 years, *n* = 8,651) and tertiary (13.2 ± 0.03 years, *n* = 3,001) education (least square means ± *SE* from the model adjusting for birth year and its interaction with parental education; see Table 1). The difference in LS means of menarcheal age between the women from highly versus poorly educated families was even larger (0.4 years) when calculated from the model adjusting for body mass and height (Supporting Information Tables S3 and S4 in ESM). Menarcheal age decreased with year of birth. The rate of this decline was substantially faster among women from families with primary parental education (slope −0.017 ± 0.002 [*SE*] years per year) than among women from families where at least one parent had tertiary education (slope −0.003 ± 0.002; see Table 1).

Rate of breast development was highest among girls from families where at least one parent was in nonmanual profession (2.23 ± 0.01 in 6‐point scale, *n* = 2,301), followed by families of skilled manual workers (2.20 ± 0.02, *n* = 1,936) and unskilled manual workers (2.14 ± 0.01, *n* = 5,094; LS means ± *SE* adjusted for age as a covariate and presented for 14.5 years old girls). We did not detect any secular change in the rate of breast development (Table 3).

Analysis of pedigree data (Table 4) revealed substantial heritabilities of menarcheal age. Additive genetic variances showed no consistent pattern with respect to parental education levels. Residual variance in menarcheal age was lowest in families of highly educated parents.

**Figure 1:**
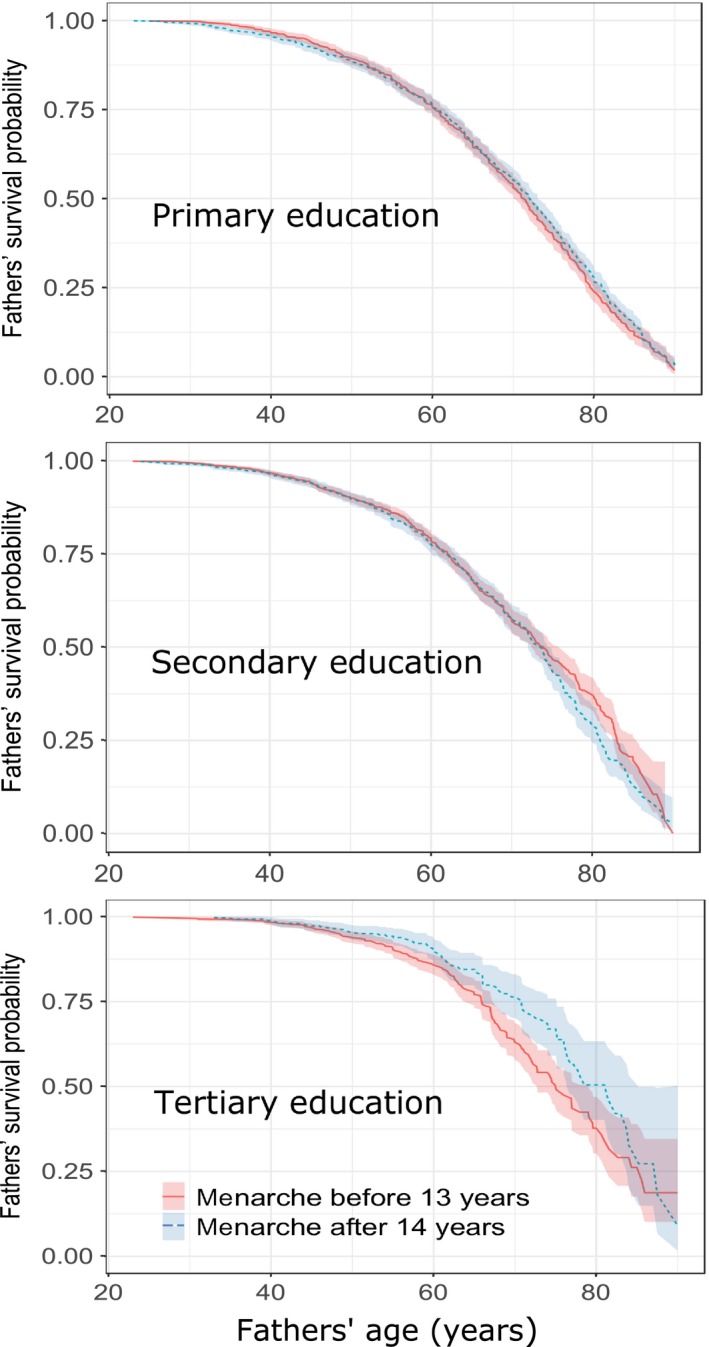
Kaplan–Meier curves for fathers’ survival probability in relation to their daughters’ age at menarche. Before 13 years = 1st quantile; after 14 years = 4th quantile. Shaded areas denote 95% CIs. Primary, secondary and tertiary education denotes highest level of education obtained by at least one parent

Daughters’ age at menarche did not predict age at death of their mothers (Tables 5A and 6A). However, it did predict survival of their fathers, but in interaction with parental education. Specifically, daughters’ menarcheal age was related to lifespan of their fathers only in families where at least one parent had tertiary education (Table 5B). In such families, 1‐year delay in daughter's menarche corresponded to 9% lower hazard of father's death (HR = 0.91, 95% CI = 0.86‐0.97, *p* = 0.004, *n* = 2 808, number of deaths = 544; see also Figure 1). Calculations using mothers’ and fathers’ education as separate predictors of parental survival did not reveal any relationships with daughters’ menarcheal age (Supporting Information Table S6 in ESM).

**Figure 2:**
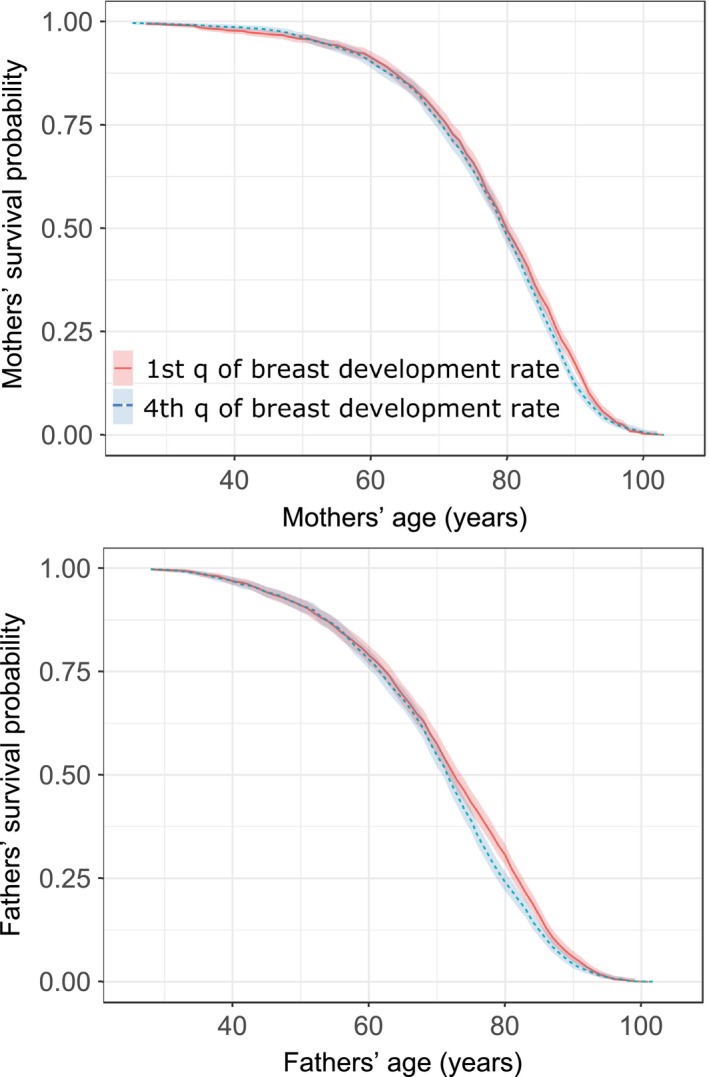
Kaplan–Meier curves for parental survival probability in relation to their daughters’ breast development rate. 1st q is 1st quantile; 4th q is 4th quantile. Shaded areas denote 95% CIs

In Aul's data set, both fathers and mothers of the girls who showed faster development of breasts died younger (Figure 2). This relationship holds irrespectively whether the model used highest parental SEP (Table 6) or mothers’ and fathers’ SEP separately (Supporting Information Table S8 in ESM) as covariate. Mothers of the girls within the highest quantile of breast development rate had 11% higher chances of death than mothers of the girls who were in the first quantile of breast development rate (HR = 1.11, 95% CI = 1.03‐1.20, *p* = 0.007; *n* = 1,346 dead and 51 alive mothers in the fourth quantile, 1,343 dead and 59 alive mothers in the first quantile). In the cases of fathers, the difference was 14% (HR = 1.14, 95% CI = 1.06‐1.24, *p* = 0.0009, *n* = 1,226 dead and 8 alive fathers in the fourth quantile, 1,212 dead and 12 alive fathers in the first quantile; see also Figure 2). None of the models revealed significant interactions between daughters’ rate of breast development and parental SEP (Table 6 and Supporting Information Table S8 in the ESM).

## 4 DISCUSSION

Results of this study show that intergenerational associations between the rate of pubertal maturation in women and longevity of their parents can be detected as phenotypic correlations, although specific patterns of this association varied between the data sets used. We will start by explaining why and how slowly maturing individuals are expected to live longer, proceed by discussing why testing the predictions of life‐history theory in our two data sets yielded different outcomes, and why the association between daughters’ rate of maturation and lifespan of their parents was expected to depend on growth conditions.

### 4.1 Why would rate of maturation associate with parental lifespan?

According to the theory of life‐history evolution, rates of maturation and reproduction of organisms should evolve to track the variation in mortality rates, so that earlier sexual maturation is favoured under high risk of early death, due to either external (Stearns, 1992) or internal (Rickard, Frankenhuis, & Nettle, 2014) causes. Eventually, such coevolution would result in genetic variants enabling longer life and those supporting slower pubertal maturation ending up in the same individuals. At the level of individuals within a population, this genetic association may (but does not need to) materialize as a phenotypic correlation between the rates of pubertal maturation and lifespan. Among women in contemporary western societies, such phenotypic correlations are frequently observed at individual level so that early menarche is typically associated with an increased risk of all‐cause mortality, ischaemic heart disease and breast cancer (reviewed by Charalampopoulos et al., 2014, the mechanisms are described by Jasienska, Bribiescas, Furberg, Helle, & Núñez‐de la Mora, 2017).

Individual‐level associations between lifespan and rate of sexual maturation may reflect either within‐population polymorphisms in antagonistic pleiotropic effects (Corbett, Courtiol, Lummaa, Moorad, & Stearns, 2018; Laisk et al., 2019) or induced phenotypic responses to womb or childhood environment (Lea, Tung, Archie, & Alberts, 2017; Wells, 2017). For instance, the risk of developing diseases related to metabolic syndrome among early‐maturing women might appear inevitable consequence of (initially adaptive) plastic response of a “thrifty phenotype” to low maternal investment *in utero* (Wells, 2017), but also result from the overlap of genomic loci associated with early menarche with genes implicated in metabolic diseases (Day et al., 2015; Perry et al., 2014). Similarly, early maturation of girls growing up without father present in the family may appear a predictive response to cues of environmental harshness or uncertainty that favours switching to a precocious life‐history strategy (Belsky, Steinberg, & Draper, 1991; Ellis, 2004) or simply a result of genetic coupling between propensity towards behaviours leading to divorce (and/or early death) and fast sexual maturation (Comings, Muhleman, Johnson, & MacMurray, 2002). Due to such ambiguities, phenotypic correlations between life‐history traits that are measured at the level of individuals are difficult to interpret in an evolutionary context (Ellis, Figueredo, Brumbach, & Schlomer, 2009). In intergenerational studies, this problem can be indirectly circumvented by taking advantage of fact that children inherit 50% of each of their parents’ genomes, so that the phenotype of offspring is used as a proxy measure of parental genotype (see Conley & Sotoudeh, 2016). For instance, measuring the rate of maturation in the offspring and age at death of their parents enables to exclude some of the potentially confounding effects of external factors that could affect the maturation and lifespan simultaneously when both are measured in the same individual.

Our results are consistent with the concept of population‐level genetic polymorphism of human life‐history traits and their combinations, although our data do not enable us to verify that rate of pubertal maturation and lifespan are genetically correlated. Age at death in humans is heritable (reviewed by van den Berg, Beekman, Smith, Janssens, & Slagboom, 2017) with genetic influence increasing with age (Giuliani, Garagnani, & Franceschi, 2018). For instance, heritabilities for surviving past 65 and 85 years in Framingham Heart Study were 36% and 40%, respectively, while the genetic effects on lifespan were found weaker prior to 60 years of age (Murabito, Yuan, & Lunetta, 2012). With epidemiologic transition in the middle of 20th century, noncommunicable diseases have become the major causes of death in the developed world, with behavioural (including lifestyle and dietary) risk factors largely responsible for the variation in the ages of death (Ezzati & Riboli, 2013). For instance, Courtenay (2000) found that leading causes of disease and death among men in USA are clearly linked to over 30 behaviours and lifestyle habits that are controllable and can be modified. Psychometric traits relating to such behaviours include impulsivity, sensation seeking and delay discounting, all of which show moderate to high heritabilities (*h* ^2^ = 0.19 − 0.80; Anokhin, Grant, Mulligan, & Heath, 2015; Harden, Quinn, & Tucker‐Drob, 2012; Li, Chen, Li, & Li, 2014; Niv, Tuvblad, Raine, Wang, & Baker, 2012) and vary across the fast–slow continuum of life‐history speed (Del Giudice, 2014). Further, the evidence is accumulating about the specific genetic variants with relatively strong effects on lifespan. For instance, various SNPs in the locus that codes nicotinic acetylcholine receptor subunits are associated with smoking quantity, nicotine and alcohol dependence, lung cancer, chronic obstructive pulmonary disease and peripheral arterial disease (Joshi et al., 2016). Another GWAS detected correlations between the genetic variants related to earlier puberty and those related to adverse health‐related outcomes, including higher BMI, type 2 diabetes, lipid profiles and cardiovascular disease (Day et al., 2015). According to the life‐history theory, selection cannot eradicate genetic variants associated with shorter lifespan if such variants are inherited together with genetic variants that increase fitness through an increased reproduction, for instance by facilitating earlier sexual maturation. One possible outcome of such process would be intergenerational correlation between the rate of pubertal maturation of daughters and lifespan of their parents, as observed uniformly in the sample of schoolgirls in Aul's database. Our findings are in particularly good agreement with those of a large study in the UK and US biobanks, showing that a higher polygenic score for the later age of menarche in daughters was associated with higher ages of death among both mothers and fathers of the participants (Mostafavi et al., 2017). The similarity of associations among both sexes is not surprising, given the strong genetic correlation (0.74) between male and female puberty timing, detected in a large GWAS (Day et al., 2015).

Would the patterns emerging in the current study be amenable to alternative explanations than a genetic correlation between lifespan and pubertal maturation rate? One of such alternatives would be population stratification, that is, nonrandom association of environments with genetic variation due to ancestry. In our case, this would mean that women inclined to early maturation are disproportionally overrepresented in areas where average lifespan is shorter. For instance, deprived neighbourhoods in the UK are characterized by low life expectancy, younger age at first birth (AFB) and higher reproductive rates (Nettle, 2010), so sampling individuals from the neighbourhoods of different quality would inevitably produce a number of correlations between life‐history traits that are consistent with the evolutionary predictions.

Ultimately, the way to exclude confounding effects due to population stratification is to analyse genetic differences between relatives who share the same ancestry (Conley et al., 2015; Gaydosh, Belsky, Domingue, Boardman, & Harris, 2018). We admit that unsuitability of our data for performing this kind of analysis is an important limitation of the current study. However, we would like to stress the important difference between the studies that use the rate of pubertal maturation versus AFB as measures of life‐history speed. Co‐occurrence of early AFB, high mortality and morbidity and steep discounting of future in low‐SEP environments is well documented (Pepper & Nettle, 2017). However, the associations between the rates of pubertal maturation and quality of the growth environment (in terms of parental SEP) are usually opposite to the associations between the SEP and AFB (Jasienska et al., 2017). Due to physiological constraints, rate of pubertal maturation is typically hindered in the low‐SEP settings, as can be seen also in the current study: menarche was latest among girls born to poorly educated parents, and the development of breasts was slowest among the daughters of unskilled manual workers. In both data sets, the lifespan of parents with poor education or low SEP was shortest. We thus consider it unlikely that nonrandom assortment of rapidly maturing women into high‐mortality environments would have caused the intergenerational correlations between the rates of pubertal maturation and parental lifespan in our study.

### 4.2 The heterogeneity of main findings

In Aul's data set of schoolgirls, daughters’ rate of pubertal maturation predicted lifespan of both their mothers and fathers irrespective of the quality of home environment, assessed on the basis of parental SEP. Among the participants of the Estonian Biobank, daughters’ menarcheal age predicted only the lifespan of fathers in well‐educated families. This difference may stem from either technical or biological causes.

Technically, the most obvious difference between the data sets is that the number of deaths, a prime determinant of the power of log‐rank tests (Hsieh & Lavori, 2000) in Aul's data set which exceeds that of the Estonian Biobank in the case of mothers (5,503 vs. 3,624) but not in the case of fathers (5,030 vs. 6,316). Lack of adequate statistical power might thus well explain why we failed to detect any associations between daughters’ menarcheal age and survival of their mothers in the biobank data. However, in the case of fathers such an explanation would not suffice. Another technical issue relates to the assessment of the quality of growth environment that was based on different proxies in the studied data sets (parental education in the data of Estonian Biobank and parental SEP in Aul's data). On the other hand, it would be difficult to imagine that SEP of the parents of schoolgirls would appear worse proxy for growth conditions than highest parental education in the data set of biobank.

Biologically, the studied data sets resemble and differ in several important aspects. Both menarcheal age and the rate of breast development reflect the pace of activation of hypothalamic–pituitary–gonadal axis and depend on the same hormones (Rapkin, Tsao, Turk, Anderson, & Zeltzer, 2006). Further, largely the same genetic effects seem to be involved in the timing of both phenotypes (van den Berg et al., 2006; Mbarek et al., 2016; Mustanski, Viken, Kaprio, Pulkkinen, & Rose, 2004). However, the few studies that have examined phenotypic correlations between these traits at individual level have found only moderate associations (Mustanski et al., 2004; Rapkin et al., 2006). One should thus not assume that the pathways linking these two markers of the rate of sexual maturation to other life‐history traits are necessarily identical. Notably, the current study also showed that although both traits showed similar dependence on the quality of home environment (assessed either on the basis of parental education or SEP), only menarcheal age revealed secular trend.

Further biological and technical differences might relate to the birth dates of the cohorts involved in the biobank and schoolgirl study. Birth years of women in the Biobank sample range from 1925 to 1996, while those of Aul's data set only span from 1936 to 1961. It is thus highly likely that shorter time span of Aul's study inevitably led to the lower variation in the growth conditions during puberty, which may explain also the absence of secular trends in the breast development rate. On the other hand, unlike in Aul's study, the participation in the Estonian Biobank was voluntary, rendering it susceptible to recruitment (Leitsalu et al., 2014) and mortality (Conley & Sotoudeh, 2016) selection. For instance, more educated, socio‐economically advantaged and healthier individuals are more likely to survive to be eligible to participate in genetic sampling in biobanks. If certain genetic profiles are associated with increased risks for early mortality, then individuals with such genotypes are less likely to be observed in older samples (Domingue et al., 2017).

Finally, it is important to notice that parents of the participants of Estonian Biobank were born on average one to two decades later than those in the schoolgirls’ study (Supporting Information Tables S1 and S2 in ESM). This means that leading causes of death are likely to differ between these cohorts, for example, due to epidemiologic transition occurring in the middle of 20th century. Even more importantly, this also means that because most of the parents of participants in Estonian Biobank are still alive, those who have died did so in relatively young age (e.g., fathers at 61 years on average). In contrast, nearly all parents of studied schoolgirls are dead, mean age at death for fathers being 74 years (see Supporting Information Tables S1 and S2 in ESM). This means that in situations where differences in parental lifespan between slowly and rapidly maturing girls do not start to emerge before age of 75 (as can be seen in Figure 2b), the chances of detection of such differences are much lower in the samples where most of parents have not yet reached that age.

### 4.3 Interaction between daughters’ menarcheal age and quality of their home environment predicts fathers’ lifespan

We predicted that the association between daughters’ rate of sexual development and parental age of death will be manifested contingent upon interaction with the quality of home environment, so that the measures of puberty predict parental lifespan most strongly in well‐off families. This prediction was based on the assumption that genetic differences in the rate of pubertal maturation are expressed most clearly among women from well‐educated families because in such families, the contribution of environmental variance to total phenotypic variance in menarcheal age is smallest. As predicted, we detected a decreasing trend in *V*_R_ with increasing parental education (Table 4); however, the Bayesian intervals for genetic parameters were wide and largely overlapped. Therefore, the hypothesis that genetic differences among individuals will be more expressed in higher quality environments is not strongly supported by these results, although the trend is in the predicted direction. Our heritability estimates for menarcheal age (*h* ^2^ = 0.56 − 0.61) are in good agreement with those obtained in previous twin and pedigree studies (Towne et al., 2005); however, such estimates are always prone to inflation due to shared environment and maternal effects (Falconer & Mackay, 1996).

The finding that fathers’ lifespan was associated with daughters’ menarcheal age only in families where at least one parent had tertiary education is thus consistent with our idea that in such families, individual values of daughters ‘menarcheal age were closer to their genetically set limits than among the girls growing up in less well‐educated families. Consistent with such an explanation is also generally earlier age at menarche in well‐educated families (Tables 1 and 2), which is indicative of relatively affluent growth conditions that reduce the proportion of environmental variance to total phenotypic variance of menarcheal age (Table 4). For instance, in such families late menarcheal age is more likely to reflect genetic predisposition to late maturation than in less well‐off families where late maturation more often results from environmental constraints—improper nutrition and/or high physical workload (see Ong et al., 2006). We thus suggest that relatively lower proportion of environmental variation in menarcheal age in well‐educated families may be the reason why daughters’ menarcheal age correlated with paternal lifespan only in such families. Such social moderation of the level of heritability has been previously demonstrated for behavioural traits, including the age at first intercourse, educational attainment, intellectual development (reviewed by Guo & Stearns, 2002) and early fertility (Kohler, Rodgers, Miller, Skytthe, & Christensen, 2006).

Admittedly, the reasoning presented above does not appear adequate for explaining the heterogeneities emerging in this study. For instance, we do not have a general explanation (besides weaker test power) for why we did not find any associations between daughters’ menarcheal age and lifespan of their mothers. Similarly, it is notable that contrary to our predictions, the association between daughters’ rate of breast development and parental lifespan was uniform in the cases of fathers and mothers and across all SEP categories. These uncertainties call for the further research on the associations between different proxies of the rate of pubertal maturation and lifespan. For instance, the associations between the rate of pubertal maturation and parental lifespan can be tested in larger biobanks than the one used in the current study. Such data sets would enable finer classification of growth environments and causes of death of participants and their parents on the basis of living conditions and epidemiological situations that differ between birth cohorts and social strata. Such intergenerational studies would offer novel opportunities for uncovering the patterns of human life‐history evolution, provided that researchers acknowledge the inherent biases, stemming from nonrandom sampling of population (Conley & Sotoudeh, 2016).

## 5 CONCLUSIONS AND IMPLICATIONS

Genomic studies have shown that genetic variants associated with early puberty are linked to those related to several disease conditions that can shorten lifespan (Day et al., 2015) and that genetic variants associated with early menarche predict shorter parental lifespan (Mostafavi et al., 2017). This study showed that phenotypic correlations between rate of pubertal maturation and lifespan can be detected at intergenerational level in two different data sets of contemporary humans. If our interpretation that these correlations reflect underlying pleiotropic associations between different components of fitness is correct, then this finding has implications for predicting the future directions of human life‐history evolution. Differential reproduction of early‐ versus late‐maturing women could affect evolution of longevity, provided that reductions of environmental variation in the rate of maturation (due to general equalization of growth conditions) expose genetic differences in menarcheal age to selection. Selection pressures favouring early maturation in women would constrain the evolution of longer lifespan of both women and men, while selection favouring late maturation would relax such constraints on lifespan. Currently, increasing proportion of world population is entering into situation where genetic differences in rates of maturation could be revealed, setting up an arena for evolutionary changes in traits genetically correlated with rate of sexual maturation.

In general, our findings are consistent with an idea of human life‐history evolution becoming more easily detectable in modern societies with individuals having more opportunities to express their genetic predispositions to different life‐history patterns (Aarssen, 2005; Bolund et al., 2015). For instance, epidemiological transition in the middle of 20th century resulted in a huge decline in mortality rates from infectious diseases in developed countries, thereby increasing the relative prominence of behavioural causes of death (Kruger & Nesse, 2006). Lifespan hence appears currently better marker of individual life‐history speed than in the pretransition period. In parallel, demographic transition was accompanied by relaxing the impact of social and normative influences, as well as harsh economic conditions on reproductive decisions of individuals, and this leads to additive genetic variation explaining more of the total variation in reproductive patterns than in the pretransition period (Bolund et al., 2015; Kohler et al., 2006). This, in turn, should make the timing of reproduction and lifetime reproductive success better markers of inherited life‐history speed than at the times when women had less control over their reproduction. Such increases in expression of genetic variation in reproductive patterns have a potential to affect the evolution of the whole suite of genetically correlated behavioural, physiological and life‐history traits.

## CONFLICT OF INTEREST

None declared.

## AUTHOR CONTRIBUTIONS

MV located the data of schoolchildren, built up and complemented the database. TK analysed pedigree data. PH and MV designed the study. PH analysed the data and wrote the manuscript. KF, RM, MV and TK contributed to data analysis and writing.
