# Supplementary material for: Parents of early‐maturing girls die younger
Source: Evol Appl. 2019 Mar 23;12(5):1050–61. doi: 10.1111/eva.12780 (PMC6503892; doi:10.1111/eva.12780)
Supplement: Supplementary file 1 [file EVA-12-1050-s001.pdf]

Table S1. Descriptive statistics for mothers' birth years and ages at death in a sample used for analysing association between daughters' menarcheal age and mothers' survival (Table 1A and B; data of Estonian Biobank).

Highest Parental Education	Mother's birth year						Mother's age at death	
	Morther alive			Mother dead				
	n	Mean (SE)	range	n	Mean (SE)	range	Mean (SE)	range
Primary	2695	1937.8 (0.2)	1914-75	1967	1924.4 (0.3)	1892-1971	71.8 (0.3)	20.6-90.9
Secondary	6571	1951.2 (0.1)	1917-74	910	1934.7 (0.4)	1901-69	63.8 (0.5)	23.8-91.0
Tertiary	2578	1952.2 (0.2)	1920-73	228	1936.9 (0.8)	1912-66	62.7 (1)	21.0-90.4
**Highest Parental Education**	**Father's birth year**						**Father's age at death**	
	Father alive			Father dead				
	n	Mean (SE)	range	n	Mean (SE)	range	Mean (SE)	range
Primary	1348	1940.0 (0.3)	1918-72	3387	1923.3 (0.2)	1875-1966	65.3 (0.2)	23.9-91.0
Secondary	5282	1951.2 (0.1)	1919-72	2388	1936.8 (0.3)	1888-1970	57.9 (0.3)	14.0-90.9
Tertiary	2264	1951.6 (0.2)	1920-74	541	1937.0 (0.6)	1900-1969	59.9 (0.6)	23.0-90.3

Table S2. Descriptive statistics for parents' birth years and ages at death in a sample used for analysing association between daughters' rate of sexual maturation (breast development in 6-point scale) and parental survival (Table 7). Parental Socioeconomic Position (SEP) is scaled as unskilled manual (1), skilled manual (2) and non-manual (3) workers (Aul's database).

Mothers' SEP	Mother's birth year						Mother's age at death	
	Morther alive			Mother dead				
	n	Mean (SE)	range	n	Mean (SE)	range	Mean (SE)	range
1	114	1928.1 (0.4)	1917-38	3945	1916.2 (0.1)	1890-1939	78.5 (0.2)	22-104
2	46	1929.8 (0.6)	1921-40	588	1920.3 (0.3)	1897-1936	79.1 (0.5)	41-106
3	81	1928.9 (0.4)	1916-38	833	1920.8 (0.2)	1898-1937	78.8 (0.4)	27-103
**Fathers' SEP**	**Father's birth year**						**Father's age at death**	
	Father alive			Father dead				
	n	Mean (SE)	range	n	Mean (SE)	range	Mean (SE)	range
1	6	1928.5 (1.2)	1925-32	2244	1912.1(0.2)	1881-1940	73.2(0.2)	28-102
2	13	1928.3 (1.4)	1921-38	1015	1917.1(0.3)	1886-1935	73.3(0.4)	28-101
3	20	1926.9 (0.8)	1921-35	900	1916.4(0.3)	1885-1935	74.3(0.4)	29-103

Table S3. Effect of highest parental education on menarcheal age of their daughters, adjusted for covariates (data of Estonian Biobank).

Effect	DF	F	p
Highest parental education	2, 17263	11.2	0.001
Height	1, 17263	275.1	<0.0001
Weight	1, 17263	415.4	<0.0001
Birth year	1, 17263	259.2	<0.0001
Highest parental education x Birth year	2, 17263	11.0	0.001

Table S4. Descriptive statistics for menarcheal age (means and least square (LS) means, adjusted for covariates in Table S3) and birth year of participants according to the highest education level obtained by their parents (data of Estonian Biobank).

	n	Menarcheal age (years)			Birth year	
Highest parental education		Mean (SE)	Range	LS Mean (SE)	Mean (SE)	range
Primary	5628	13.60 (0.02)	8-19	13.47 (0.03)	1959.8 (0.16)	1925-93
Secondary	8644	13.28 (0.02)	9-19	13.31 (0.02)	1974.9 (0.13)	1931-95
Tertiary	2999	13.15 (0.02)	9-19	13.12 (0.03)	1977.8 (0.19)	1929-96

Table S5. Estimates of the heritability and variance structure of menarcheal age in relation to the highest parental education level in the family, calculated from the animal model accounting for the fixed effect of birth year, using package VCE 6.0.2. V_A, additive genetic variance; V_R, residual variance, which includes environmental effects, nonadditive genetic variance (dominance and epistatic), and error variance; V_P, phenotypic variance (data of Estonian Biobank).

Highest parental education	N	Mean (SD)	h^2(SE)	V_A(SE)	V_R(SE)	V_P
Primary	5 628	13.60 (1.52)	0.555 (0.082)	1.263 (0.190)	1.014 (0.187)	2.278
Secondary	8 651	13.28 (1.41)	0.543 (0.065)	1.071 (0.131)	0.902 (0.127)	1.973
Tertiary	3 001	13.15 (1.33)	0.584 (0.140)	1.043 (0.255)	0.743 (0.246)	1.786

Table S6. Cox proportional hazard models for survival of mothers and fathers in relation to menarcheal age of their daughters and its interaction with education level of mother and father (data of Estonian Biobank).

A. Mothers' survival, n = 8 708, number of deaths = 1 011		
Predictor	Hazard ratio (95% CI)	P
Mother's year of birth	1.045 (1.036-1.055)	<0.0001
Daughters' menarcheal age (A)	1.030 (0.972-1.090)	0.299
Mother's education is secondary (B)*	2.098 (0.551-7.993)	0.278
Mother'seducation is tertiary (C)*	0.767(0.046-12.869)	0.854
Father's education is secondary (D)*	0.641 (0.163-2.520)	0.525
Father's'education is tertiary (E)*	3.268 (0.207-51.504)	0.400
AxB	0.929 (0.841-1.026)	0.146
AxC	0.977 (0.789-1.210)	0.831
AxD	1.036 (0.936-1.146)	0.498
AxE	0.899 (0.729-1.109)	0.322
B. Fathers' survival, n = 8 539, number of deaths = 2 167		
Fathers' year of birth	1.045 (1.039-1.051)	<0.0001
Daughters' menarcheal age (A)	1.007 (0.966-1.050)	0.729
Mother's education is secondary (B)*	0.754 (0.316-1.798)	0.525
Mother'seducation is tertiary (C)*	1.705 (0.301-9.674)	0.547
Father's education is secondary (D)*	0.411 (0.316-1.016)	0.054
Father's'education is tertiary (E)*	0.890 (0.142-5.581)	0.901
AxB	1.015 (0.951-1.082)	0.661
AxC	0.939(0.824-1.071)	0.348
AxD	1.045 (0.977-1.118)	0.199
AxE	0.958 (0.951-1.082)	0.549

*compared with mothers or fathers from families where either mother or father had primary education.

Table S7. Cox proportional hazard models for survival of mothers and fathers in relation to breeding values of menarcheal age of their daughters and its interaction with highest parental education level in the family. In families comprising sisters, all but one sister is randomly excluded (data of Estonian Biobank).

A. Mothers' survival, n = 14 621, number of deaths = 3 125		
Predictor	Hazard ratio (95% CI)	P
Mothers' year of birth	1.027 (1.023-1.032)	<0.0001
Daughters' menarcheal age (A)	1.002 (0.974-1.031)	0.900
Highest parental education is secondary (B)*	0.668 (0.327-1.366)	0.269
Highest parental education is tertiary (C)*	1.137 (0.297-4.351)	0.851
AxB	1.006 (0.955-1.060)	0.822
AxC	0.949 (0.857-1.051)	0.316
B. Fathers' survival, n = 14 456, number of deaths = 6 039		
Fathers' year of birth	1.010 (1.007-1.012)	<0.0001
Daughters' menarcheal age (A)	0.994 (0.971-1.017)	0.611
Highest parental education is secondary (B)*	0.536 (0.326-0.879)	0.0134
Highest parental education is tertiary (C)*	1.422 (0.581-3.482)	0.441
AxB	1.029 (0.992-1.067)	0.126
AxC	0.922 (0.861-0.987)	0.019

*compared with parents from families where both parents had primary education.

Table S8. Cox proportional hazard models for survival of mothers and fathers in relation to the rate of sexual maturation (age-standardized breast development score) of their parents and their own SEP (Aul's database).

A. Mothers' survival, n = 5 607, number of deaths = 5 366		
Predictor	Hazard ratio (95% CI)	P
Mothers' year of birth	1.006 (1.010-1.017)	0.001
Daughters' breast development score	1.050 (1.012-1.093)	0.011
Mother skilled manual*	0.839 (0.768-0.916)	<0.0001
Mother non-manual*	0.833 (0.768-0.912)	<0.0001
B. Fathers' survival, n = 4 198, number of deaths = 4 159		
Fathers' year of birth	1.014 (1.007-1.011)	<0.0001
Daughters' breast development score	1.077 (1.031-1.125)	0.0009
Father skilled manual*	0.883 (0.818-0.953)	<0.0001
Father non-manual*	0.763 (0.705-0.827)	<0.0001

*compared with parents on non-skilled manual professions. Interaction terms between breast development score and parental SEP were non-significant both in the case of mothers (HR = 1.081 (0.949-1.231) and 1.007 (0.903-1.232)) and fathers (HR = 1.103 (0.992-1.226) and 1.012 (0.905-1.315)).

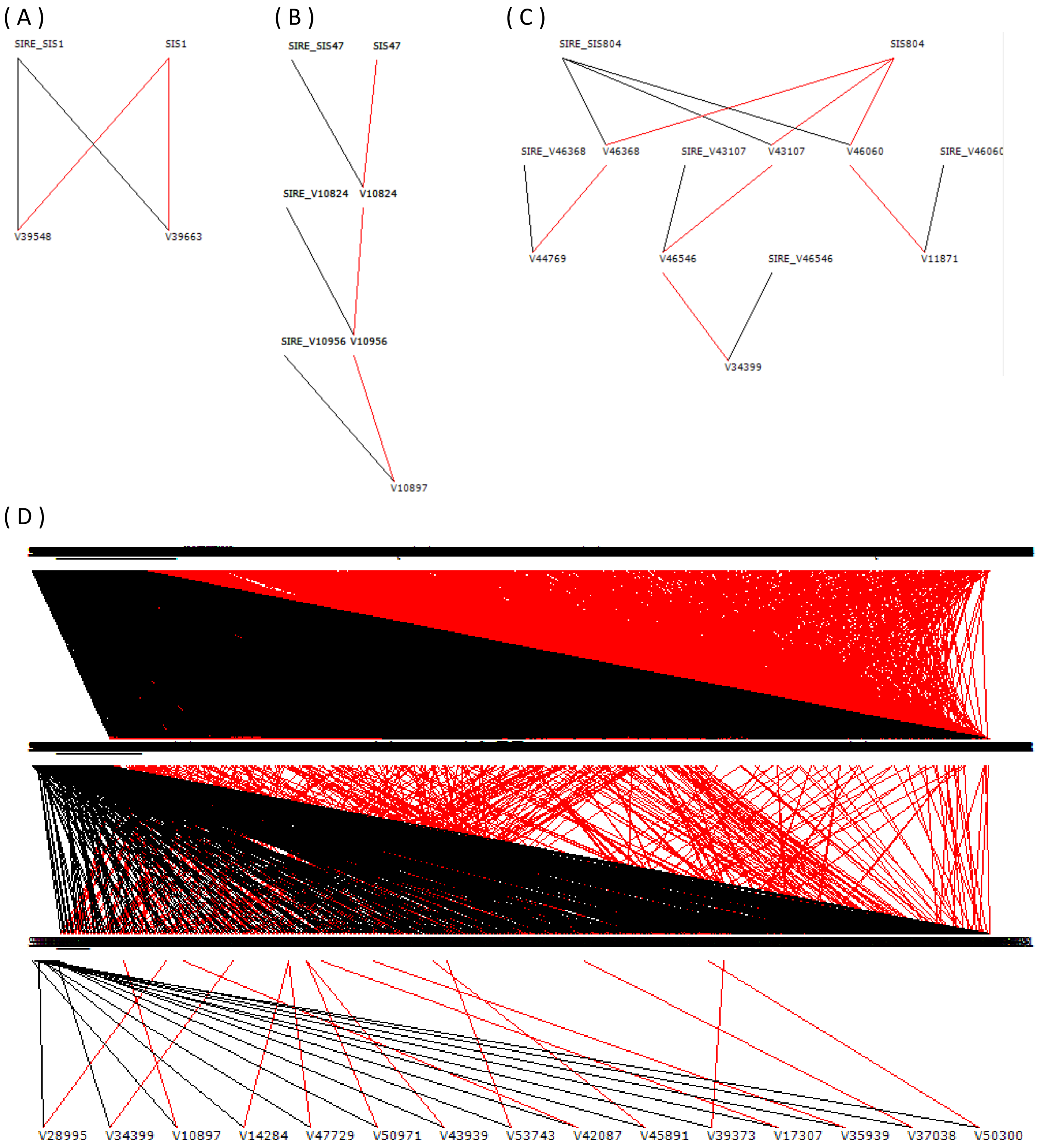

Figure S1. A, B, C – examples of different pedigree-based relationships, red and black lines indicate mother and father, respectively, identification codes starting with capital ‘V’ indicate women with known menarcheal age; D – whole pedigree used in animal model analyses.
